# Endothelial cellular senescence and tau accumulation: An interplay full of opportunities?

**DOI:** 10.1002/ibra.12154

**Published:** 2024-05-09

**Authors:** Doriana Oliveri, Giorgia Moschetti, Anna Griego, Edoardo Scarpa

**Affiliations:** ^1^ Department of Pharmaceutical Sciences (DISFARM) University of Milan Milan Italy; ^2^ Infection Dynamics Laboratory‐National Institute of Molecular Genetics (INGM) Milan Italy

**Keywords:** brain, cellular senescence, tauopathies

## Abstract

Recent research has shown that tau protein can be passed to neighboring cells, leading to cellular senescence in the endothelial cells present in the central nervous system (CNS). This discovery could potentially open new doors for testing novel therapeutic compounds that specifically target senescent cells (senolytics) or for identifying new biomarkers that can enable early detection of tauopathies and dementia.

## Graphical abstract

Recent research has shown that tau protein can be passed to neighbouring cells, leading to cellular senescence in the endothelial cells present in the central nervous system (CNS). This discovery could potentially open new doors for testing novel therapeutic compounds that specifically target senescent cells (senolytics), or for identifying new biomarkers that can enable early detection of tauopathies and dementia.

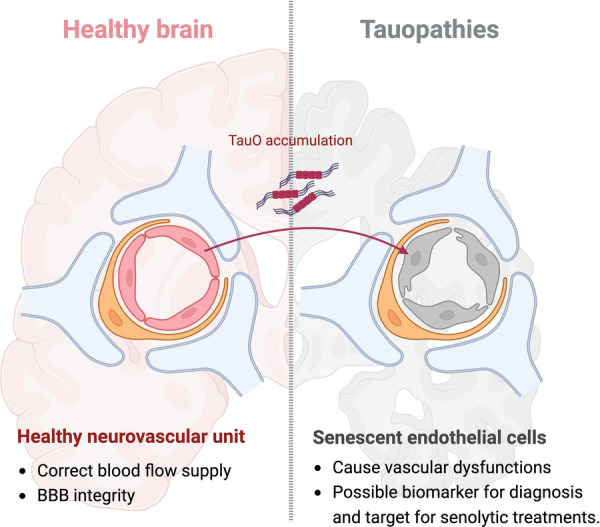

## 1 INTRODUCTION

Tauopathies are a group of neurodegenerative diseases, which include Alzheimer's disease (AD), marked by abnormal accumulation of tau protein. They are known to be the leading cause of dementia.^1^ These disorders can cause a wide range of debilitating symptoms, including impaired functions of movement and language, as well as cognitive decline and amnesia episodes.^2^ Still today, tauopathies remain incurable, and current therapeutic interventions only address symptoms.^3^

## 2 TAU OLIGOMER ACCUMULATION AND NEURODEGENERATION

**Figure 1:**
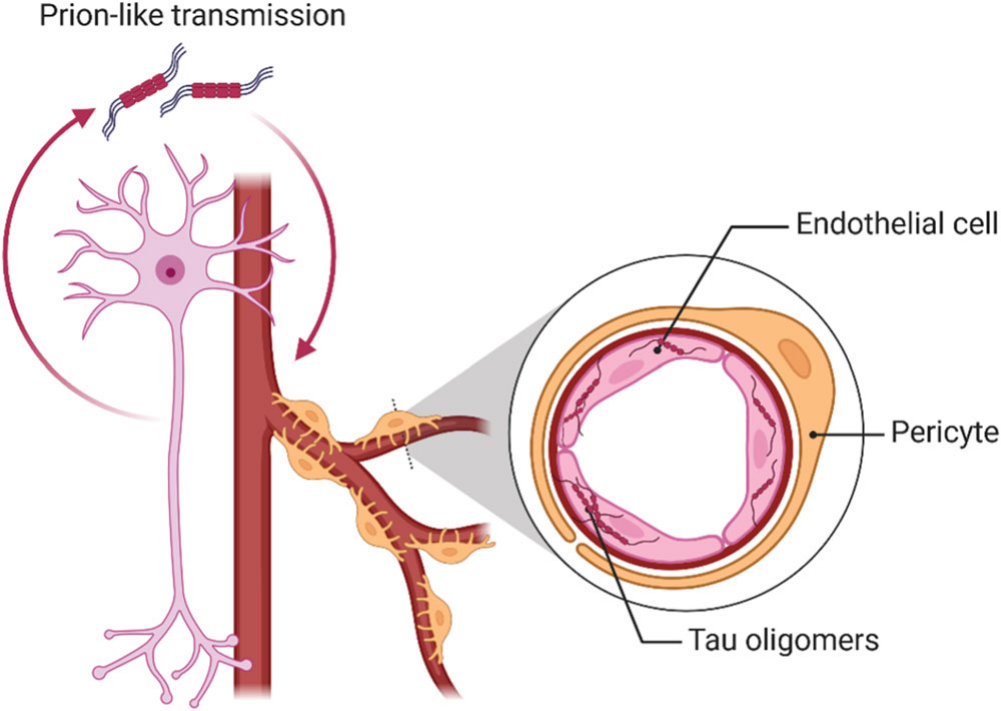
Trans‐neuronal propagation of TauO in a prion‐like manner. Tau misfolded proteins exhibit a prion‐like transmission, disseminating among neurons. TauO can also be internalized by endothelial cells, where they contribute to senescence induction, which alters cell functionality, and to the propagation of neurodegeneration. TauO, tau oligomers. [Color figure can be viewed at wileyonlinelibrary.com]

The prevalence of tau deposition has been classically regarded as an appealing molecular target for interventions.^4^ Nevertheless, the poorly understood etiology and pathology driving the neurodegenerative process resulted in the lack of effective treatments. Physiologically, tau protein binds to microtubules, determining their stability. However, in tauopathies, tau protein accumulates in its misfolded forms, including the pathogenic soluble tau oligomers (TauO) and the insoluble neurofibrillary tangles (NFT).^5^ Both TauO and NFT accumulation have been associated with cognitive decline and synaptic impairment, but TauO is the most cytotoxic form. Differently from NFTs, TauO can propagate trans‐neuronally through the extracellular milieu, resembling a prion‐like mechanism^6^ (Figure 1). Once in the intracellular compartment, the misfolded structure of exogenous TauO affects the endogenous tau pool and leads to the propagation of its pathogenic form and, thus, to the progression of the disease.^7^ Pathological tau deposits are classified as primary tauopathies and are considered sufficient to drive neurodegeneration. However, tau accumulation can be followed by the aggregation of a second protein (secondary tauopathies), like beta‐amyloid plaques in AD, which accelerates the pathology.^8^ For example, tau accumulation in memory‐related regions correlates with dementia progression, and it is a predictor of overall severity.^5^ A crucial question regarding tau accumulation is its potential to spread in different anatomical regions within and between tissues.

## 3 IMPACT ON BRAIN MICROVASCULATURE

New studies have recently demonstrated that TauO accumulation is not limited to neuronal cells, but it also extends to endothelial cells, affecting the brain microvasculature and causing functionality deficits.^7^ Endothelial cells, together with pericytes and astrocytes, constitute the neurovascular unit, guaranteeing the correct blood flow supply and the integrity of the blood–brain barrier (BBB). However, a loss of pericytes, altered functionality of endothelial cells, and the consequent reduction of blood flow have been observed in patients affected by tauopathies.^6^ Overall, vasculature dysfunctions occur in the early stage of the disease, even before the clinical manifestation, and they impact disease progression. However, the underlying molecular mechanism leading to cognitive decline still needs to be better characterized.^9^ An in‐depth characterization of the effects that tau accumulation has on neuronal and bystander cells could lead to the discovery of early biomarkers of pathology and potential new therapies.

In this context, the molecular drivers and cause‐effect play between TauO deposition and the early appearance of vasculature dysfunctions have recently been clarified.^9^, ^10^ New evidence from Hussong et al. demonstrated that TauO can spread in a prion‐like manner in the cerebral region, hence reaching the brain microvasculature, where TauO binds the endothelial cell via heparan sulfate proteoglycans and is internalized by micropinocytosis affecting the cytoskeleton organization.^7^, ^11^ The authors demonstrated the mechanism mediating TauO internalization both in vitro, using human brain microvascular endothelial cells (HBECs), and in vivo, employing a mouse model for tauopathies PS19Tg (MAPT P301S). In addition, they verified the correlation between TauO accumulation and vasculature dysfunction through decreased nitric oxide (NO) production. NO is an essential regulator of endothelial‐dependent brain vascular dilatation. Its production occurs via endothelial nitric oxide synthase (eNOS), which requires microtubule structure to reach the membrane compartment for its activation.^7^ The authors showed that an impaired translocation of eNOS in cells resulted in TauO accumulation and thus induced microtubule dysfunctions.^7^ The impact of TauO on the pathogenesis of tauopathies is thus not only circumscribed to neurons, but it propagates to other cell types, like brain endothelial cells. Indeed, eNOS impairment could also occur in other key players of the neurovascular unit (e.g., astrocytes and pericytes) that regulate access to and from the brain. Furthermore, considering that vasculature dysfunctions appear in the early stages of the disease, TauO is an effective biomarker to detect the onset of tauopathies and prevent the progression of cognitive decline. For example, the levels of hyperphosphorylated tau (p‐tau) in plasma have been investigated as a potential blood‐based biomarker for the early detection of AD and demonstrated tangible clinical promises.^12^ On the other hand, the selective elimination of TauO might represent an effective target for potential therapy aiming at impeding the dissemination of misfolded tau proteins and the subsequent formation of NFTs. While numerous anti‐tau antibodies underwent clinical trials, their inability to progress to phase III primarily stemmed from a lack of specificity, inadvertently targeting both cytotoxic and nonpathogenic forms of tau protein.^5^, ^13^ In this regard, the exploration of tau oligomer‐specific monoclonal antibodies to remove pathogenic soluble aggregated tau in mice models of tauopathies showed the ability to ameliorate microvasculature dysfunction.^7^ Despite these promising developments, it is crucial to acknowledge that these findings are in their preliminary stages. Consideration of potential peripheral side effects of the antibody and a comprehensive evaluation of its impact on cognitive functionalities remain vital aspects to be thoroughly explored. Nonetheless, these results open the possible direct translation of this therapy to treat, at least, early‐stage tauopathies.

## 4 CELLULAR SENESCENCE: BETWEEN BIOMARKER AND THERAPY

**Figure 2:**
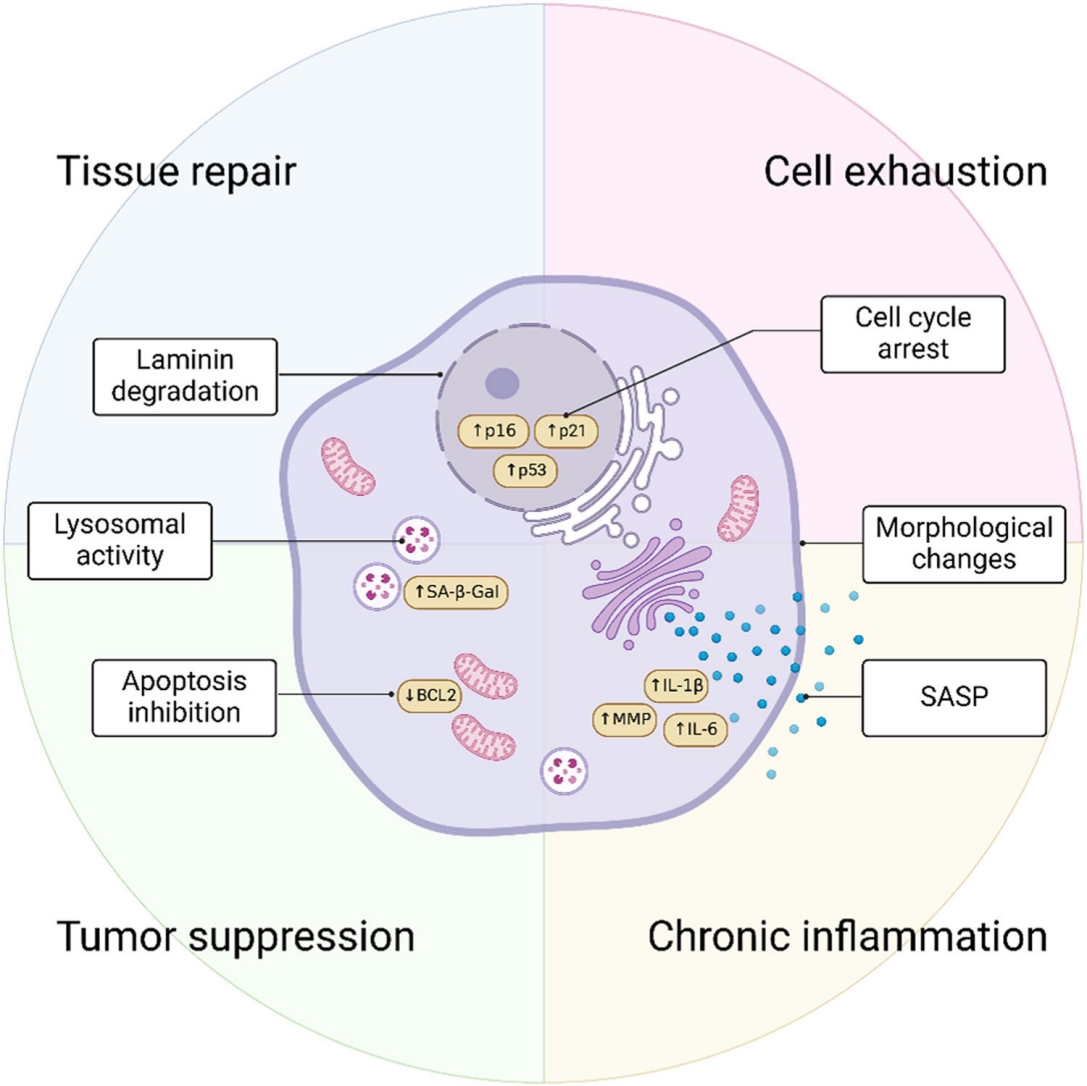
Cellular senescence. Senescent cells display morphological changes including cell flattening and enlargement and laminin degradation. These cells exhibit high resistance to apoptosis and cell‐cycle arrest. However, senescent cells remain metabolically active as they increase SA‐β‐Gal production and SASP, which promote an inflammatory environment and senescence transmission. Functionally, senescent cells play both beneficial and detrimental roles. SASP, senescence‐associated secretory phenotype; SA‐β‐Gal, senescence‐associated beta‐galactosidase. [Color figure can be viewed at wileyonlinelibrary.com]

Another intriguing aspect investigated by Hussong and colleagues is that the accumulation of TauO in endothelial cells determines not only a functional impairment but also a phenotypic transition and the onset of cellular senescence.^7^ Cellular senescence is characterized by high resistance to apoptosis and the arrest of the cell cycle, together with morphological and metabolic changes^14^ (Figure 2). On one hand, cellular senescence can be beneficial, for example, to promote tissue repair after injury or to inhibit cancer cells over‐proliferation.^14^, ^15^ On the other hand, it can be detrimental as senescent cells decrease their functionality and become hypersecretory to promote a proinflammatory environment that is classically referred to as the senescence‐associated secretory phenotype (SASP).^16^, ^17^ Accumulation of senescent cells occurs across various tissues with age as a normal physiological process, resulting in the continuous release of SASP factors, which contribute to inflammaging, a chronic inflammatory environment associated with aging that fosters disease and dysfunction.^5^ In the context of brain health and neurodegeneration, there is a need to thoroughly understand the role of senescent cells and their implications. Novel and exciting investigations are populating the literature elucidating the interconnection between senescence‐associated features in different cell types, aging, and neurodegenerative disorders (recently reviewed^18^). In this context, it was demonstrated that the entry and accumulation of TauO, but not tau monomers, was sufficient to trigger senescent‐like features in HBECs and brain microvascular isolates from PS19Tg (MAPT P301S) mice. These cells were characterized by upregulation of cell‐cycle inhibitors (cyclin‐dependent kinase inhibitor 1 A [CDKN1A], cyclin‐dependent kinase inhibitor 2 A [CDKN2A], tumor protein P53 [TP53]) together with genes related to SASP such as interleukin‐1β and interleukin‐6, albeit with different secretory signatures.^7^ It was suggested that microtubule destabilization caused by TauO accumulation and concurrent eNOS reduction could promote senescence. It is intriguing to explore the possible connection between the destabilization of endothelial microtubules and a senescence‐like state.^7^ However, it is crucial to determine whether this connection is the root cause of senescence or a mere consequence. Research shows that the accumulation of TauO starts in neurons, and patients with AD have a subset of excitatory neurons that exhibit senescence‐associated features, especially cyclin‐dependent kinase inhibitor 2D (CDKN2D/p19).^19^ These senescent neurons are similar to those containing NFT, thus connecting AD with cellular senescence. Therefore, it is challenging to dismiss the hypothesis that these cells could induce cellular senescence in the endothelial bystander cells through the secretion of SASP, in a process commonly referred to as “secondary senescence.” Overall, these observations prompt questions about whether these senescent features actively contribute to AD pathology and tauopathies or if they arise as a consequential aspect of the pathological process.

Studies using the classical transgenic mice p16Ink4a+ have suggested the causative role of senescent cells in promoting the progression of neurodegenerative diseases.^20^ Clearly, it is not surprising that the targeting of senescent cells could constitute another potential therapy to prevent cognitive decline. Senotherapeutics are a class of molecules that specifically target senescent cells. They are classically divided into senolytics, which selectively eliminate senescent cells or induce senolysis, and senomorphics, which attenuate the production and release of SASPs. While senolytics promote apoptosis by targeting enzymes involved in pro‐survival and antiapoptotic mechanisms (such as Bcl‐2 family proteins, Akt, phosphoinositide 3‐kinase [PI3K]), senomorphics suppress senescence by suppressing SASP expression via targeting nuclear factor‐kappaB (NF‐κB), mechanistic target of rapamycin (mTOR), interleukin‐1α (IL‐1α), mitogen‐activated protein kinase (p38 MAPK), and other signalling pathways.^21^ Studies conducted on mice models of tauopathy have shown that senescent cells can be cleared pharmacologically to reduce brain pathogenesis. In comparison to mice treated with a placebo, intermittent administration of the two senolytics, dasatinib and quercetin, resulted in a reduction of tau accumulation and neuroinflammation. Their combined use helped preserve neuronal and synaptic density, restored aberrant cerebral blood flow, and alleviated cognitive deficits.^4^, ^22^, ^23^, ^24^ Intermittent dosing of dasatinib and quercetin showed an acceptable safety profile in clinical studies for other senescence‐associated conditions.^4^, ^22^, ^23^, ^24^ Along these premises, a feasibility phase I study on senolytic therapy comprising these two compounds in mild AD has just been completed.^25^ Overall, the results demonstrated a safe profile of the therapy, and although the study was not designed to evaluate the efficacy, the data suggest the potential for treatment‐related changes in markers of cellular senescence and AD pathology. Notably, quercetin was not detected in the cerebrospinal fluid of the patients, differently from dasatinib, which might indicate the inability of this molecule to cross BBB.^25^ The efficacy of senolytic compounds in treating age‐related neurodegenerative diseases depends on their ability to permeate the BBB and reach target cells in the brain. Although novel senotherapies are under development, in vitro studies and tests in preclinical models should be conducted to determine whether these therapies can effectively penetrate the BBB and access senescent cells in the central nervous system.^26^, ^27^ It is important to note the discrepancy between studies reporting that both dasatinib and quercetin could cross the BBB and the phase I clinical trial findings in which quercetin was not detected in the CNS.^25^ BBB permeability can be impaired by pre‐existing pathological conditions, and the fact that tau can affect the brain microvasculature is a clear example. Thus, it is plausible that further investigations will confirm the ability of senotherapeutics to accumulate in the brains of patients with tauopathies. Another compelling question is whether the pan elimination of the senescent cells would actually be more beneficial than detrimental. Thus far, all the senolytics generated are completely unspecific and are unable to discriminate subpopulations of senescent cells. Clearing senescent cells in peripheral tissues may also positively impact the brain,^28^ but developing delivering strategies promoting both BBB crossing and specific targeting is desirable. Already existing examples of nanoparticles are able to efficiently cross the BBB to deliver therapeutic compounds^29^, ^30^, ^31^, ^32^ and potentially target cellular subpopulations.^33^ However, the combination of senolytics with these nanoparticles is yet to be explored.

## 5 FUTURE PERSPECTIVES

Moving forward, it is logical to consider tauopathies, and neurodegenerative diseases in general, as multifaceted pathologies that concern multiple cellular factors, either in a direct or indirect fashion. The emergent link between neurodegeneration and cellular senescence is providing novel investigative avenues. Nonetheless, long‐teasing questions require further investigations to push the field forward. Can we exploit cellular senescence as a biomarker for early detection? It appears that features of senescence are present in the brain even before the onset of the classical symptoms of neurodegeneration.^18^ Could the detection of cellular senescence in particular regions of the brain, perhaps through SASP characterization, support the detection of blood p‐tau in AD?

To answer this question, it would be imperative to identify senescence‐associated features of diverse brain cell types and different pathological conditions. The recent emergence of single‐cell sequencing techniques and proteomic analysis, especially with spatial resolution, is expected to aid in identifying specific senescence markers and characterizing the composition of SASP for various cell types and pathologies.^34^

This would allow not only to stratify cellular senescence according to its function within the brain tissue but also to generate a roadmap to guide therapeutic interventions. Such investigation would also answer the question “Can we precisely eliminate only specific senescent cells?”. Indeed, generating a barcode of pathologic senescent cells will provide a platform for the generation of targeted therapies that could circumvent the off‐target issues associated with senotherapies. In conclusion, tauopathies are characterized by a microenvironment where cellular senescence might play a crucial role in driving the progression of the disease. Hence, targeting those cells might represent an alternative and effective strategy for developing therapies that could save millions of lives.

## AUTHOR CONTRIBUTIONS

Doriana Oliveri wrote and edited the manuscript and prepared the figures. Giorgia Moschetti and Anna Griego reviewed the manuscript. Edoardo Scarpa wrote and/or edited the manuscript before submission.

## CONFLICT OF INTEREST STATEMENT

The authors declare no conflict of interest.

## ETHICS STATEMENT

Not applicable.

## Data Availability

Not applicable as the manuscript does not contain novel data, and all the information provided can be sourced from literature.
